# Immunological Consequences of *In Utero* Exposure to Foreign Antigens

**DOI:** 10.3389/fimmu.2021.638435

**Published:** 2021-04-15

**Authors:** Jeng-Chang Chen

**Affiliations:** Department of Surgery, Chang Gung Children’s Hospital, College of Medicine, Chang Gung University, Taoyuan, Taiwan

**Keywords:** fetus, *in utero* exposure, sensitization, hematopoietic chimerism, macrophage, bone marrow transplantation, alloantigen, immune tolerance

## Abstract

Immunologic tolerance refers to a state of immune nonreactivity specific to particular antigens as an important issue in the field of transplantation and the management of autoimmune diseases. Tolerance conceptually originated from Owen’s observation of blood cell sharing in twin calves. Owen’s conceptual framework subsequently constituted the backbone of Medawar’s “actively acquired tolerance” as the major tenet of modern immunology. Based upon this knowledge, the delivery of genetically distinct hematopoietic stem cells into pre-immune fetuses represented a novel and unique approach to their engraftment without the requirement of myeloablation or immunosuppression. It might also make fetal recipients commit donor alloantigens to memory of their patterns as “self” so as to create a state of donor-specific tolerance. Over the years, the effort made experimentally or clinically toward *in utero* marrow transplantation could not reliably yield sufficient hematopoietic chimerism for curing candidate diseases as anticipated, nor did allogeneic graft tolerance universally develop as envisaged by Medawar following *in utero* exposure to various forms of alloantigens from exosomes, lymphocytes or marrow cells. Enduring graft tolerance was only conditional on a state of significant hematopoietic chimerism conferred by marrow inocula. Notably, fetal exposure to ovalbumin, oncoprotein and microbial antigens did not elicit immune tolerance, but instead triggered an event of sensitization to the antigens inoculated. These fetal immunogenic events might be clinically relevant to prenatal imprinting of atopy, immune surveillance against developmental tumorigenesis, and prenatal immunization against infectious diseases. Briefly, the immunological consequences of fetal exposure to foreign antigens could be tolerogenic or immunogenic, relying upon the type or nature of antigens introduced. Thus, the classical school of “actively acquired tolerance” might oversimplify the interactions between developing fetal immune system and antigens. Such interactions might rely upon fetal macrophages, which showed up earlier than lymphocytes and were competent to phagocytose foreign antigens so as to bridge toward antigen-specific adaptive immunity later on in life. Thus, innate fetal macrophages may be the potential basis for exploring how the immunological outcome of fetal exposure to foreign antigens is determined to improve the likelihood and reliability of manipulating fetal immune system toward tolerization or immunization to antigens.

## Introduction

Immunological tolerance refers to a state of immune nonreactivity specific to particular antigens as the holy grail in the field of transplantation and autoimmune diseases. The concept of tolerance germinated in 1945 when Ray Owen discovered blood cell chimerism in dizygotic twin calves with confluent fetal circulations *via* unique placental vascular anastomoses (1). Owen’s observation guided the researchers to experimentally demonstrate reciprocal tolerance toward skin grafts of chimeric dizygotic twins (2, 3) and paved the way to the formulation of “actively acquired tolerance” in 1953 by Peter Medawar, who preemptively inoculated murine fetuses with a mixture of donor strain cells (4). Medawar’s work represented a conceptual advance of immune tolerance. By extension, it was deemed to provide experimental support for Burnet’s theory that self-nonself discrimination by the immune system was not genetically programmed but rather gradually learned in the embryonic period or immediately post-embryonic stages (5). In other words, antigen exposure before full immune development might elicit tolerance to this specific antigen, making the intrauterine life favorable for the implementation of medical interventions that will be later hampered by immune responses. Based upon Medawar’s knowledge and approach, prenatally-induced immune tolerance has been experimentally replicable, but never been a universal event in all or even most subjects of analogous experiments by many researchers and our team. Paradoxically, the event of *in utero* immunization to foreign antigens might be sometimes experienced. Thus, a thorough review of the experimental work involving fetal exposure to foreign antigens might highlight the inconsistent and even conflicting outcomes, and help to clarify the debates on this topic.

## Fetal Tolerance to Maternal Antigens at the Maternal-Fetal Interface

During pregnancy, placentation allows an intimate contact between maternal and fetal cells at the maternal-fetal interface, where bidirectional exchange of both mature and progenitor cells occurs (6, 7). This two-way cell traffic contributes to fetal cells in mothers (fetal microchimerism) and maternal cells in offspring (maternal microchimerism), which play a pivotal role in averting maternal-fetal immunological conflict during pregnancy (8). Although such microchimerism was reportedly associated with the pathogenesis of autoimmune diseases (9), a large body of studies showed that developmental exposure to non-inherited maternal antigen (NIMA) in the form of maternal microchimerism would be of benefit to the outcome of NIMA-matched transplants (7, 10, 11). This tolerizing effects of maternal microchimerism on fetal immune system, termed as the “NIMA effect” (7), are essentially compatible with the principle of Medawar’s “actively acquired tolerance”, whereas possible mechanisms might involve not only the central deletion of NIMA-reactive T cells but also the induction of peripheral regulatory T-cells (7, 11). Of note, the NIMA effect was highly relevant to the degree of maternal microchimerism (12–14).

## Historical Review of Tolerance Induction After *In Utero* Exposure to Foreign Antigens

The concept of “actively acquired tolerance” has fascinated immunological community for more than half a century and attracted a number of laboratory work to replicate this immunological phenomenon (15). In the 1950s, neonatal exposure to soluble proteins such as ovalbumin, bovine serum albumin, or human albumin and gamma globulin was claimed to cause immunological unresponsiveness to these peptide antigens (16–18). Although these animal studies at first glance seemed to mirror Medawar’s work, an in-depth review for their approaches to immune tolerance revealed that they might not be always conducted or analyzed in a scientifically sound and sophisticated way. For example, tolerance to soluble peptide antigens was defined simply by either delayed clearance of antigens injected (17, 18), or decreased percentage of fatal anaphylaxis to antigen re-challenge without considering the underlying mechanism behind the shock in individual animals (16).

During the 1960s, cells of different tissue origins were examined for their tolerance-conferring capacity (19). Nodal or splenic lymphocytes were found to have the excellent tolerogenic ability to render the immunologically immature neonates tolerant of skin allografts (19, 20). In A (H-2K^k^, D^d^, L^d^, I-A^k^, I-E^k^) strain murine recipients, not only did CBA (H-2K^k^, D^k^, L^null^, I-A^k^, I-E^k^) lymphocytes compare favorably in CBA skin tolerance induction with CBA×A F1 hybrid lymphocytes, but also made A strain recipients susceptible to graft-versus-host disease (GVHD) (19). Thus, graft-versus-host effects of CBA lymphocytes due to disparate H-2D and L loci between CBA and A mice should have been considered immunologically relevant to the suppression of host immunity against donor skin grafts. Subsequently, CBA lymphocytes were reported to induce CBA skin tolerance without the occurrence of GVHD in C3H (H-2K^k^, D^k^, L^null^, I-A^k^, I-E^k^) recipients (20). Immunologically, CBA skin tolerance in the absence of adverse GVHD might result from the sharing of all the H-2 loci between CBA donors and C3H recipients rather than tolerance-conferring capacity of CBA lymphocytes. In fact, the relatively weak or absent major histocompatibility complex (MHC) barriers of the two stain combinations could not reflect the reality in clinical arena with almost fully MHC-mismatched transplants. Reappraisal of the tolerogenic properties of fully MHC-mismatched naive lymphocytes revealed that fetal recipients usually succumbed to GVHD before skin tolerance could be examined (21). Therefore, the claimed superiority of allogeneic lymphocytes for tolerance induction apparently overlooked their detrimental graft-versus-host effects that might lessen donor graft rejection.

**Figure 1:**
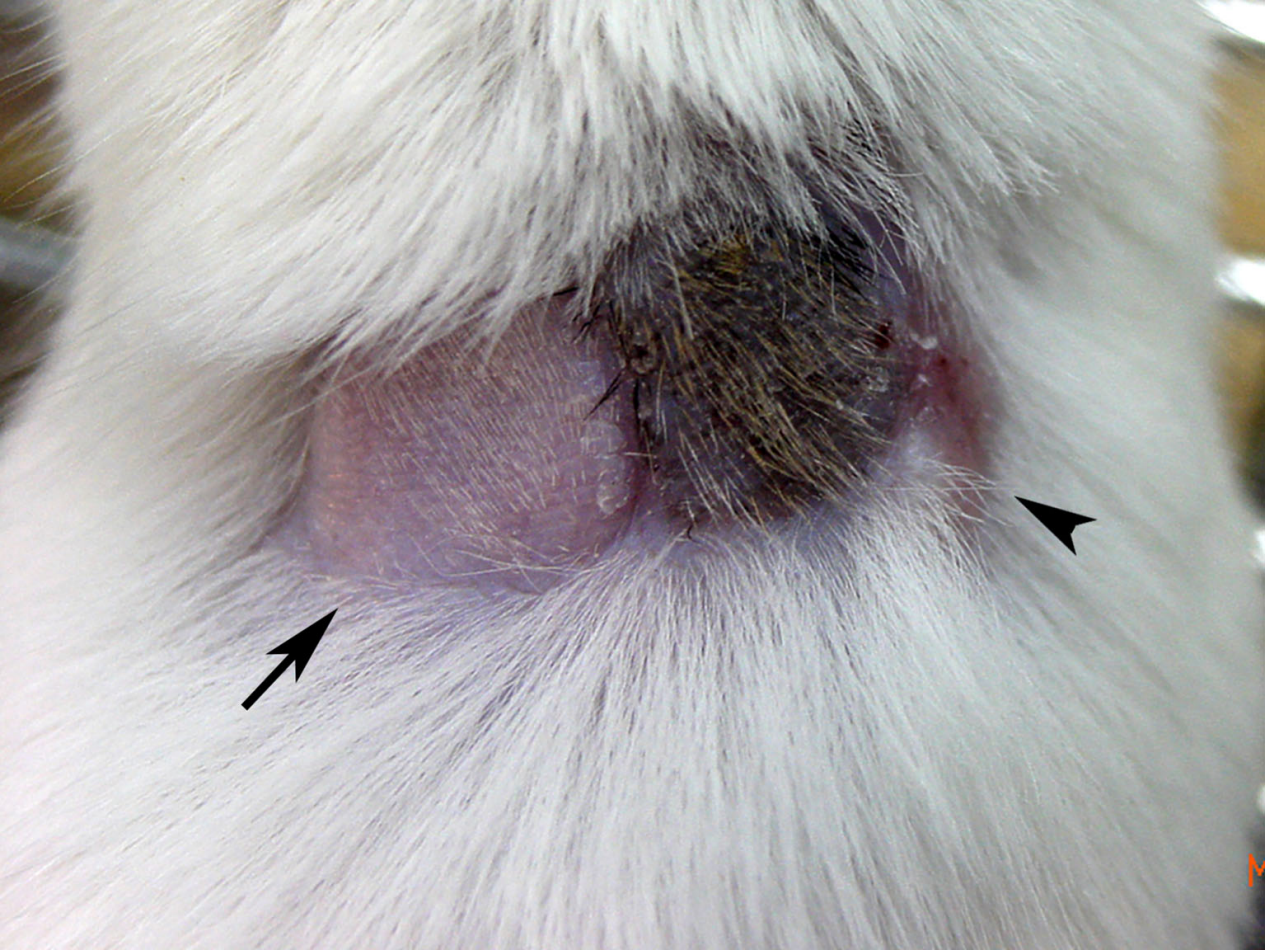
Skin graft tolerance in a state of hematopoietic chimerism. Following *in utero* injection of C57BL/6 (H-2^b^) BMCs into gestational day 14 FVB/N (H-2^q^) fetuses, skin transplantation was performed in a representative mixed chimera with 5.63% peripheral blood cell chimerism at 1 month old. The image taken at 5 months old showed that syngeneic FVB/N (arrow) and donor C57BL/6 (black hair) skin grafts were well accepted with good hair growth, but third-party C3H (H-2^k^, arrowhead) skin had been rejected with a scar. It supported a state of donor-specific immune tolerance.

From the viewpoint of modern immunology, these 1950s-60s studies of immune tolerance indeed left something to be desired. Their experimental approaches and analyses reflected the scarcity of immunological knowledge about graft rejection, and limited laboratory tools at that time to investigate such a complex phenomenon of immune tolerance. In 1970s-80s, a series of studies demystified the crucial role of MHC in mediating cellular immunity (22) and enabling transplantation rejection (23, 24). Following the discovery of T-cell receptors (25) and a clear understanding of T-cell ontogeny in respect of T-cell receptor development (26, 27), immunology had already advanced considerably to an extent that could not be envisaged in 1950s-60s. In 1995, Carrier et al. (28) reassessed *in utero* tolerance induction in a fully MHC-disparate nondefective murine model (**Table 1**). Gestational days 11-13 fetuses were subjected to the injection of fetal liver hematopoietic stem cells (HSCs) early before the emergence of T-cells with T-cell receptor expression (on around gestational day 17) (26). Postnatally, enduring donor skin tolerance only developed in 3 (14%) of 22 fetal recipients. Carrier’s result was discouraging because most of fetal recipients failed to accept donor skin persistently within the terms of the experiment. In 1996, Hajdu et al. achieved prenatally-induced tolerance as donor skin acceptance only in 5 (5.1%) of 99 recipients surviving *in utero* injection of allogeneic fetal liver HSCs (30). In Kim’s series (31), 6 (20%) out of 30 prenatally-injected mice with skin transplant were tolerant to donor skin. Likewise, our team suffered from frustration at a low success rate of persistent donor skin tolerance (**Figure 1**), accounting for 25.9% (38 cases) of 147 murine recipients surviving fetal injection (32). Therefore, it was hard to reconcile a far less than 50% success rate of donor graft tolerance induction with the concept of “actively acquired tolerance”.

**Table 1:** Immunological outcome of fetal exposure to foreign antigens in mice.

Antigen type	Inocula	Recipients	Outcome	Remarks
Alloantigen (4)	Cells from testis, kidney and spleen of A strain mice (H-2K^k^, D^d^, L^d^, I-A^k^, I-E^k^)	GD_15-16_ CBA fetuses (H-2K^k^, D^k^, L^null^, I-A^k^, I-E^k^)	Skin tolerance (77, 91 and 101 days) in 3 of 6 recipients (one-set experiment)	Pioneer work of a partially MHC-mismatched model by Medawar et al. Skin tolerance > 30 days in 6/77 (15 sets of experiments) (29). No chimerism examined
Alloantigen (28)	B6 (H-2^b^) fetal liver HSCs	GD_11-13_ BALB/C (H-2^d^) fetuses	Skin tolerance >20 weeks in 3/22 recipients	Blood or tissue microchimerism
Alloantigen (30)	C3H (H-2^k^) fetal liver HSCs	GD_14-16_ B6 fetuses	Skin tolerance in 5/99 recipients	Blood microchimerism
Alloantigen (31)	B6 adult BMCs	GD_13-16_ BALB/C fetuses	Skin tolerance >8 weeks in 6/30 recipients	Blood microchimerism
Alloantigen (32)	B6 adult BMCs	GD_14_ FVB/N (H-2^q^) fetuses	Skin tolerance >120 days in 38/147 recipients	The requirement of a threshold chimerism level to establish rather than maintain postnatal skin tolerance
Alloantigen (33)	BALB/C MHC exosome or enriched B6 B cells	GD_14_ FVB/N fetuses	Decreased alloreactivity of recipient lymphocytes	Delayed skin rejection in a state of B cell microchimerism
Alloantigen (34)	B6 adult c-kit^+^ cells	GD_12-13_ BALB/C fetuses	In utero sensitization	Accelerated skin rejection with alloreactivity in MLR. Microchimerism might lead to alloreactivity.
Alloantigen (35)	B6 adult Sca^+^Lin^-^ cells	GD_13_ BALB/C fetuses	*In utero* sensitization	No chimerism detected by PCR, Anti-donor alloreactivity in MLR
Alloantigen (36)	B6 adult Sca^+^Lin^-^ or c-kit^+^Lin^-^ cells	GD_11-13_ BALB/C fetuses	*In utero* sensitization	Accelerated skin rejection with enhanced cytotoxicity and Th1 cytokine release in Sca group
Allergen (37)	Ovalbumin, Derp II	GD_14_ FVB/N and BALB/C fetuses	*In utero* sensitization	Allergen contact in pre-immune fetuses might initiate Th2-skewed atopy to facilitate allergy development.
Oncoprotein (38)	HPV E7	GD_14_ FVB/N and B6 fetuses	*In utero* sensitization	Fetuses could mount Th1 tumoricidal immunity against tumorigenesis following in utero exposure to tumor antigens.
Microbial antigen (39)	Salmonella antigens	GD_14_ FVB/N fetuses	*In utero* sensitization	Fetuses were competent to mount adaptive immunity to microbial antigens and defend against the pathogens in postnatal life.

GD, gestational day; MLR, mixed lymphocyte reaction; PCR, polymerase chain reaction; Derp II, Dermatophagoides pteronyssinus group II; HPV, human papillomavirus; HSC, hematopoietic stem cell; BMC, bone marrow cell.

In 2018, Medawar’s work was illuminated from a distinctive perspective by Hyung Wook Park (29). It was found that Medawar did not publish the whole story of his experimental tolerance induction in murine fetuses. A review of Medawar’s laboratory notes revealed that tolerance recorded as their donor skin survivals of over 1 month merely happened to 6 of 77 fetal recipients in 15 sets of experiments (a partially MHC-mismatched model, **Table 1**), along with failure or breakdown of skin grafts at median survival time of 11 days in 56, survival for 12-14 days in 4, and for 15-30 days in 11 (29). The overall success rate was estimated at 7.8% (6/77), far much lower than formally reported in 1953 Nature manuscript (3 out of six murine fetuses, Exp. 73) (4). The whole picture called into question as to whether such a low success rate of Medawar’s experiment sufficed to be the decisive and conclusive evidence supporting Burnet’s theory of self-nonself discrimination (15), and reflect the reality of fetal exposure to foreign antigens.

## Hematopoietic Engraftment After *In Utero* HSC Transplantation

Taking advantage of the pre-immune windows as proposed by Medawar, *in utero* HSC transplantation stands out as a promising approach to hematopoietic engraftment across allogeneic barriers without the requirement of myeloablative or immunosuppressive regimens (40). As a result, it was once deemed as a substitute for postnatal bone marrow transplantation to cure a variety of genetic disorders such as immunodeficiencies, hemoglobinopathies and inborn errors of metabolism before disease onset in postnatal life (41). However, over the past few decades, the clinical progress on *in utero* marrow transplantation has never been made in parallel with the fast-moving advances in prenatal diagnosis, fetal intervention, and stem cell technology. Its clinical application was precluded by the difficulty in consistently achieving sufficient levels of allogeneic hematopoietic engraftment, as evidenced by limited success only in inherited immunodeficiency diseases, but little or even no clinical benefit to congenital hematological or metabolic disorders (40, 41).

It was reported that the therapeutic benefit to murine (42) or human (40) beta-thalassemia by cellular therapies necessitated donor cell chimerism of 10-20%. Animal studies of *in utero* HSC transplantation, aimed to artificially replicate blood chimerism, had been conducted in sheep (43), monkeys (44), swine (45), canine (46–48) and mice (28, 32, 49, 50). However, hematopoietic engraftment was usually unsatisfactory, far below that expected of therapeutic significance at the levels of 10-20% except for fetal sheep and sporadic recipients in canine and murine models. The sheep model represented a rare but remarkable success in fetal transplantation. Three of 4 normal fetal lambs developed donor cell chimerism of 14-29% following fetal peritoneal injection of HSCs (43). In canine fetuses, therapeutically significant chimerism (>10%) could be achieved by intravascular administration of HSCs (46), in sharp contrast to low-level chimerism by intraperitoneal approach (47, 48). However, HSC injection *via* portal vein of fetal swine only led to microchimerism (45).

The mouse has been the most popular model for the studies of *in utero* HSC transplantation, whereas hematopoietic reconstitution that reached therapeutic significance before 2000 mainly succeeded in genetically anemic (51, 52) or immunodeficient (53, 54) murine fetuses. In normal murine fetuses, transplants with graded doses of light-density bone marrow cells (BMCs) containing 1-2% CD3 T-cells showed a dose response in the chimerism rate and level (49). High-level chimerism (>10% donor cells) emerged with a threshold dose of 5×10^6^ BMCs administered to gestational day 14 murine fetuses. At the dose of 7.5-10×10^6^, high-level chimeras accounted for around 14% of fetal recipients surviving the injection and exhibited multilineage hematopoietic reconstitution. However, high-level chimerism achieved by T-cell containing marrows was accompanied by an over 50% incidence of GVHD (49). Depletion of marrow T-cells prevented GVHD but lessened hematopoietic engraftment (49, 55–58). It was worth mentioning that developing fetuses were even more vulnerable to allogeneic T-cell attack, as evidenced by the observation that fully MHC-mismatched lymphocytes rapidly elicited lethal GVHD in fetal recipients (21). As a result, the benefit from allogeneic T-cells in hematopoietic reconstitution must be weighed against the potential risk of GVHD in pre-immune fetuses.

As for naturally-occurring hematopoietic engraftment in dizygotic twins of cattle, sheep, goats (59), primates (60) and even humans (61, 62), its levels were reported to range from micro- to nearly full chimerism, similar to what had been observed following artificial administration of allogeneic marrows *in utero* (32, 49). The inconsistent engraftment even happened to a litter of pups injected *in utero* with an identical dose of marrow inocula from the same batch (49). The hidden obstacles to allogeneic HSC engraftment during gestation periods might comprise maternal T-cells (63), the competitive milieu (64, 65) and potential immune barriers (66, 67) of fetal recipients. However, it remained difficult to explain why high-level donor cells could persist only in peritoneum where marrow cells were inoculated prenatally (49, 68). Although *in utero* HSC transplantation was not considered to be reliably therapeutic, it might be alternatively employed to prenatally induce donor-specific tolerance, thereby mitigating or even obviating the allogeneic immunoreactivity to facilitate postnatal therapies (50).

## Allogeneic Graft Tolerance in a State of Hematopoietic Chimerism

Skin allograft rejection was first described as a phenomenon of immune reactions in a burn patient and the rabbit model in 1940s (69, 70). Subsequently, it was found that this immune defense against allografts could be mitigated or even blocked in a state of hematopoietic chimerism in twins with naturally-occurring blood exchange (1-3) or through artificial inoculation of mixed donor cells during pre-immune fetal stage (4). Similar graft tolerance could be observed in a state of peripheral leukocyte chimerism following donor marrow cell infusion in an irradiated adult dog preparatory to renal transplantation (71). However, among a number of successful organ transplants in humans, some recipients might eventually experience immunosuppressive-free graft acceptance without preceding donor leukocyte/marrow infusion or overt donor leukocyte chimerism in circulation (72). It caused the dismissal of a link between organ engraftment and donor leukocyte chimerism for almost three decades. It was not until 1992, when Starzl et al. discovered a trace of donor leukocytes (microchimerism) in the tissues or blood of long-surviving human liver or kidney allograft recipients due to advancement in phenotyping techniques, that the association between chimerism and graft tolerance attracted attention (73, 74).

Although immunological tolerance achieved by solid organ or bone marrow transplantation shared a common phenomenon of hematopoietic chimerism, whether the chimerism had the nature in common or two distinct states remained a matter of debate. Hematopoietic chimerism after organ transplantation resulted from passenger leukocytes that previously harbored within the donor grafts and migrated ubiquitously in the recipients through circulation (73, 74). Chimeric donor cells were obviously sparse in the recipients (75), but might exhibit striking biological effects far exceeding its number (76). The tolerogenic capacity of an organ basically reflected its comparative content of migratory leukocytes, evidenced by the observation that passenger leukocyte-rich liver had much more potent tolerogenicity for its own acceptance than the leukocyte-poor kidney and heart (75).

Although Starzl’s discovery spotlighted chimerism in the transplantation community, but a consensus on the role of chimerism in graft tolerance was never reached (77). Recipients with long-term renal graft survivals might experience a low incidence (one-third) of microchimerism (78). Moreover, organ recipients with persistent microchimerism might not be weaned from immunosuppressives, or at times experienced graft rejection (79, 80). Paradoxically, the intragraft passenger leukocytes were once regarded as the major allo-immunogenic stimulus to elicit rejection (81). The inconsistency or confliction made it difficult to establish clinical benefit from microchimerism as a reliable marker of graft tolerance for discontinuing immunosuppressives in organ recipients (77, 82). It was worth mentioning that organ recipients generally demanded sufficient immunosuppressive regimens to prevent graft rejection, and as such to suppress clearance of passenger leukocytes that egressed from organ transplants to allow the occurrence of microchimerism and then the development of drug-free tolerance in some cases (75). Under the circumstances, the detection of microchimerism in the recipients did not necessarily imply the cause of graft acceptance, but might contrarily represent the result of graft acceptance or the effect of the immunosuppression required for preventing rejection (77).

Graft tolerance could be induced through the creation of mixed chimerism by bone marrow transplantation involving myeloablation and immunosuppression to various degrees (83). Years of effort with adult animal studies showed that chimerism levels achieved were directly associated with the probability or degree of graft tolerance (84–86). Recipients usually developed high-level chimerism (86–89), which was a sure warrant of donor graft tolerance (85) unless there was a lack of donor T-cell engraftment after transplantation (90, 91). However, it was difficult to evaluate to what extent donor cell levels (77) or test graft survivals (84) came from the confounding effects of preconditioning or immunosuppressive programs. Moreover, questions remained as to whether chimerism led to tolerance or an induced state of tolerance permitted chimerism (84).

The conferment of skin tolerance in Medawar’s experiment of a partially MHC-mismatched murine model was not an all-or-none event but rather a graded phenomenon (29) with test graft survivals varying from only a few days of grace beyond the median survival time to 1 month or longer. The graded phenomenon of skin tolerance was also observed following *in utero* transplantation of fully MHC-mismatched HSCs in mice (28, 32, 49), as evidenced by a wide variability of donor skin survivals, ranging from prolonged for a few days or weeks over their counterpart controls to persistent for more than 4 months, highly relevant to hematopoietic chimerism actually obtained (32, 49, 92). In the fetuses with twin-twin transfusion, naturally-occurring chimerism caused a variable degree of tolerance to reciprocal skin grafts in dizygotic twins (2, 3). It might reflect a wide range of hematopoietic chimerism caused by twin-twin transfusion (59–62), similar to what was illustrated by artificial mixing of allogeneic HSCs through *in utero* transplantation (32, 49). Thus, *in utero* HSC transplantation would be an ideal model to interpret the influence of hematopoietic chimerism on graft tolerance without the interference from myeloablation and immunosuppression.

## Induction and Maintenance of Graft Tolerance Following *In Utero* HSC Transplantation

In Hayashi’s study of fetal transplantation in mice, 1-2% circulating donor cells at 3 weeks old sufficed to sustain donor skin tolerance for 8 weeks (50). However, chimerism levels of <2% at 1 month old were not considered as durable, and might fade away by 6 months old (32). In these low-level mixed chimeras, the timing of skin grafting influenced graft acceptance with the critical parameter being the chimerism level at skin placement in preference to a higher level earlier in life prior to skin grafting (32). Thus, induction of complete skin tolerance appeared feasible within a window of opportunity afforded by the presence of sufficient circulating donor cells at skin transplantation. Donor skin tolerance consistently developed for at least 4 months with chimerism levels of >3% at skin transplantation, but appeared in a gray-zone success of around 35% with chimerism levels of 0.2-3% (32). The gray-zone chimerism levels linked to the ambiguity in predicting graft tolerance might reflect the conflicting association between tolerance induction and low-level chimerism in previous studies (28). Regardless of skin tolerance status, prenatally-created chimerism could attenuate or abolish donor-specific T-cell alloreactivity in mixed chimeras (32). Therefore, complete skin tolerance might develop through the tolerogenic effects of donor skin under a state of chimerism-related immunosuppression of host lymphocytes. Namely, hematopoietic chimerism exerted immunomodulatory effects on the induction phase of allograft tolerance. It was essentially in keeping with the proposition that inhibition of initial graft rejection by sufficient immunosuppression might allow the tolerogenic properties of organ allografts to eventually prevail (93), and also reflected the phenomenon that transfusion or adoptive transfer of donor leukocytes with solid organ transplants induced prolonged allograft survivals instead of long-term graft tolerance (94, 95).

Whether maintenance of graft tolerance relies upon chimerism remains a matter of debate. In adult recipients with skin tolerance after marrow transplantation, artificial elimination of engrafted donor cells led to the rejection of existing donor skin grafts, indicating the requirement of chimerism for tolerance maintenance (96, 97). However, it was likely that the antibodies used to deplete donor cells might jeopardize donor skin survivals. In nonhuman primates or humans treated with simultaneous marrow and renal transplants (98–100), graft tolerance persisted despite spontaneous loss of peripheral chimerism. Thus, alloantigens present in the form of the surviving organ grafts might help to maintain tolerance (83, 98, 100). In fetal recipients receiving marrow transplantation without any preconditioning, spontaneous regression of peripheral and tissue chimerism did not cause the rejection of donor skins engrafted previously under sufficient peripheral chimerism, nor did the removal of engrafted donor skin break the state of tolerance in tolerant mice that had lost peripheral chimerism (32). It argued against the necessity of donor cell chimerism or donor skin alloantigens for enduring allogeneic tolerance. Thus, prenatally-created hematopoietic chimerism was a simple and straightforward marker to predict the establishment rather than maintenance of postnatal graft tolerance.

## *In Utero* Exposure to Alloantigens in Various Forms

HSCs in BMC inocula contributed to blood cell chimerism in fetal transplantation, and stood out as being particularly pertinent to donor skin tolerance in postnatal life. Lymphocytes, rich in alloantigens but devoid of HSCs, were used as the substitute for BMCs to scrutinize the immunological outcome of prenatal alloantigen exposure independently of HSC engraftment (21). They showed lethal graft-versus-host effects on the recipients early in fetal or neonatal life, and lacked substantial capacity of conferring significant hematopoietic chimerism and skin graft tolerance even at acceptable doses. MHC exosomes and B-cells represented soluble and cellular forms of alloantigens, respectively. Their tolerogenic capacity in pre-immune fetuses was examined without the interference from HSC engraftment or graft-versus-host effects (33). Their injection *in utero* led to the suppression of host lymphocyte alloreactivity specific to donor alloantigens rather than donor skin tolerance (**Table 1**). Although highly enriched B-cell inocula might generate low-level B-cell chimerism, they only extended the survivals of donor skin grafts by a few days in the recipients.

Apparently, BMCs were the unique alloantigen inocula for *in utero* induction of allo-tolerance, which ensued conditionally on the establishment of significant hematopoietic chimerism. Despite that the dose of self-antigens determined the consequence of deletional tolerance (101) and donor T-cell engraftment were critical for tolerance induction in mixed chimeras (90, 91), neither an increase in BMC doses nor donor T-cell contents benefited skin graft survivals unless it had substantially improved peripheral chimerism following *in utero* marrow transplantation (49). Altogether, it might dawn on researchers in the field that hematopoietic chimerism had an indispensable role in facilitating skin graft survivals, arguing against the self-nonself concept that a simple contact with alloantigens early *in utero* made them perceived as self by the fetus.

## Perverse Outcome of *In Utero* Exposure to Foreign Antigens

**Figure 2:**
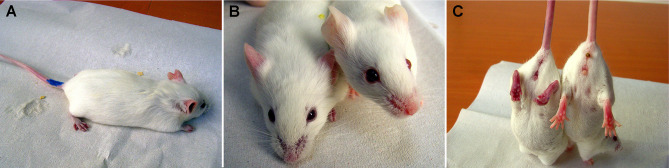
Anaphylaxis in FVB/N mice prenatally exposed to ovalbumin. Gestational day 14 FVB/N fetuses were intraperitoneally exposed to free ovalbumin peptides. Postnatally, they received intraperitoneal ovalbumin re-challenge. **(A)** Within 10-15 minutes, mice developed limb weakness and **(B)** cyanotic nose, ears, **(C)** genitalia, feet and tail as compared with the normal control (right mouse). Subsequently, the mice presented with shallow breathing, and finally succumbed to anaphylaxis. Postnatal anaphylaxis indicated a prior sensitization event in fetal life.

In the literature (**Table 1**), there was no shortage of animal studies that failed to induce allo-tolerance or oppositely initiated an event of immunization in fetal (34–36) as well as neonatal recipients (102). These inconsistent or even conflicting results seemingly clouded the picture of fetal or neonatal tolerance induction (103, 104). Armed with the knowledge of “actively acquired tolerance”, we had conducted a study to evaluate the feasibility of allergen desensitization through intraperitoneal exposure to soluble ovalbumin allergens in pre-immune murine fetuses. It turned out to be an unintended consequence of *in utero* sensitization (37), characterized by heightened recall Th2-skewed immunity and fatal anaphylaxis (**Figure 2**) in response to postnatal ovalbumin re-encounter in postnatal life. Moreover, postnatal aerosolized ovalbumin stress elicited allergic lungs, leading to functional and structural alterations of airways. Thus, fetal immunogenic capacity had the important implication for prenatal imprinting of atopy. Recently, we further disclosed that *in utero* exposure to oncoprotein triggered antigen-specific Th1 adaptive immunity to protect from tumorigenesis (38). It suggested the capacity of fetal immune system for immune surveillance against developmental tumorigenesis given an encounter with tumor antigens egressing during embryogenesis. Moreover, fetal exposure to microbial antigens could confer antigen-specific adaptive immunity against lethal microbial challenge (39), indicating the feasibility of fetal immunization against infectious diseases. As a whole, it threw into sharp relief the fact that fetal exposure to foreign antigens did not always induce tolerance, but might lead to immunogenic events with biological significance.

## The Role of Fetal Macrophages in Shaping Fetal Immune Responses

In a state of prenatally-created hematopoietic chimerism, the mechanisms underlying donor-specific T-cell nonreactivity were mainly attributed to the central deletion of donor reactive lymphocytes *via* direct or indirect pathways of antigen presentation (105, 106) despite that peripheral mechanisms of anergy and regulatory T-cells had been reported (105). Thymic deletion highly related to peripheral chimerism (105), which showed a linear correlation with thymic chimerism (32). It reflected not only the requirement of sufficient intrathymic donor cells for effective clonal deletion (105), but also the rationale for peripheral chimerism levels as a biomarker of postnatal skin tolerance (32). Thus, antigen presenting cells, either donor or recipient origin, might be critical for the immunological outcome following *in utero* exposure to foreign cells. Fetal macrophages were dendritic cell progenitors (37), emerging early during embryogenesis as the first immune cells capable of taking up nonself antigens, particles or dead cells in fetal life (107, 108). The pre-immune stage, usually referring to the period before full development of adaptive (T-cell) immunity, may not be early enough for the fetus to be “tricked” into ignoring nonself antigens as long as innate phagocytes remain functioning well and competent. Fetal macrophages were demonstrated to play a critical role in dealing with antigens present *in utero* and effectively retaining their memory after antigen internalization early before T-cell maturation so as to regulate the immunological outcome of fetal antigen exposure (37, 38). The role of fetal macrophages in clonal deletion or tolerance induction to alloantigens awaits further experimental elucidations.

## Conclusion

The immunological consequences of fetal exposure to foreign antigens were more intricate than first envisaged by Medawar. Fetal exposure to alloantigens might lessen or abolish recipient lymphocytes’ alloreactivity, but not necessarily confer donor graft tolerance. The successful induction of long-lasting postnatal graft tolerance was relatively a rarity, which constituted the course of Medawar’s experiment from the outset (29) and a number of analogous studies later as well (28, 30–32, 49, 50). More specifically, complete graft tolerance was only conditional on the achievement of significant hematopoietic chimerism following *in utero* transplantation of marrow inocula (32). Thus, allo-tolerance could not be induced simply by an early *in utero* contact with alloantigens. Contrary to “actively acquired tolerance”, *in utero* exposure to soluble ovalbumin (37), oncoprotein (38), or microbial antigens (39) triggered antigen-specific adaptive immunity. The immunogenicity in fetal life has a noteworthy relevance to human health such as prenatal initiation of allergy, immune surveillance against developmental tumorigenesis and prenatal immunization against infectious diseases. Taken as a whole, the immunological consequences of fetal exposure to foreign antigens could be tolerogenic or immunogenic, relying upon the type or nature of antigens introduced.

## Author Contributions

The author confirms being the sole contributor of this work and has approved it for publication.

## Funding

This work was supported by the grants CMRPG3G1501-3 and CMRPG3K0431-2 from Chang Gung Medical Foundation, Taiwan and the grant 109-2314-B-182-041-MY3 from the Ministry of Science and Technology, Taiwan.

## Conflict of Interest

The author declares that the research was conducted in the absence of any commercial or financial relationships that could be constructed as a potential conflict of interest.

## Abbreviations

NIMA, non-inherited maternal antigen; GVHD, graft-versus-host disease; MHC, major histocompatibility complex; HSC, hematopoietic stem cell; BMC, bone marrow cell.
